# The Dual Role of the Plastid Terminal Oxidase PTOX: Between a Protective and a Pro-oxidant Function

**DOI:** 10.3389/fpls.2015.01147

**Published:** 2016-01-05

**Authors:** Anja Krieger-Liszkay, Kathleen Feilke

**Affiliations:** Institute for Integrative Biology of the Cell, Centre National de la Recherche Scientifique, Comissariat à l'Energie Atomique et aux Energies Alternatives Saclay, Institut de Biologie et de Technologie de Saclay, Université Paris-SudGif-sur-Yvette, France

**Keywords:** plastid terminal oxidase, reactive oxygen species, abiotic stress, photosynthetic electron transport, regulation

The plastid terminal oxidase (PTOX) is a non-heme diiron quinol oxidase that oxidizes plastoquinol and reduced O_2_ to H_2_O. PTOX was discovered in the so-called *immutans* mutant of *A. thaliana* showing a variegated phenotype (Wetzel et al., 1994; Carol et al., 1999). PTOX is localized in the non-appressed regions of the thylakoid membrane (Lennon et al., 2003) and is involved in carotenoid biosynthesis, plastid development, and chlororespiration. Reviews have focused on the role of PTOX in chlororespiration (Bennoun, 2002; Rumeau et al., 2007), in chloroplast biogenesis (Putarjunan et al., 2013) and in stress responses (McDonald et al., 2011; Sun and Wen, 2011). A recent review by Nawrocki et al. (2015) has addressed the role of PTOX in poising the chloroplast redox potential in darkness. However, its role and interplay with the photosynthetic electron flow remains unclear.

Plants grown in moderate light under non-stress conditions have low PTOX concentrations (about 1 PTOX protein per 100 PSII; Lennon et al., 2003). By contrast, elevated PTOX levels have been found in plants exposed to abiotic stresses such as high temperatures, high light and drought (Quiles, 2006), salinity (Stepien and Johnson, 2009), low temperatures and high intensities of visible (Ivanov et al., 2012), and UV light (Laureau et al., 2013). PTOX has been proposed to act as a safety valve by protecting the plastoquinone pool from overreduction under abiotic stress. A highly reduced PQ pool hinders forward electron flow and triggers charge recombination in photosystem II (PSII) leading to the generation of triplet chlorophyll and highly toxic singlet oxygen. However, overexpression of PTOX in *A. thaliana* did not protect against light-induced photodamage (Rosso et al., 2006) and even enhanced photo-oxidative stress in tobacco expressing, in addition to its endogenous enzyme, either PTOX from *A. thaliana* (Heyno et al., 2009) or PTOX1 from *C. reinhardtii* (Ahmad et al., 2012). Different to higher plants *C. reinhardtii* possesses two isoforms, PTOX1 and PTOX2. PTOX1 is most likely responsible for regenerating PQ for phytoene desaturation and shows a lower rate of plastoquinol oxidation during photosynthesis than PTOX2 (Houille-Vernes et al., 2011).

Using purified PTOX, Yu and coworkers have recently shown that depending on the quinol concentration PTOX can act as an anti-oxidant or pro-oxidant (Feilke et al., 2014; Yu et al., 2014). PTOX activity was found to be pH insensitive between pH 6.0–8.5 when as substrate decylPQH_2_ dissolved in methanol was used (Yu et al., 2014). During the catalysis, peroxide intermediates are formed at the diiron center. Depending on the lifetime of these intermediates, reactive oxygen species (ROS) can be generated as a side reaction. Isolated PTOX generates superoxide radicals at both high, but physiologically relevant, quinol concentrations at pH 8.0 and substrate limiting concentrations at pH 6.0–6.5 (Feilke et al., 2014; Yu et al., 2014). When substrate is limited, the second quinol may not arrive in time leading to superoxide formation directly at the catalytic center. Alternatively, since at pH 8.0 the semiquinone is more stable than at pH 6.0, it is conceivable that the high pH stabilized semiquinone acts as a ROS generator. PTOX in overexpressors has also been found to generate superoxide in the light (Heyno et al., 2009).

By oxidizing plastoquinol PTOX reduces the number of electrons available for photosynthetic electron flow. It is generally accepted that PTOX has low activity compared to photosynthetic electron flow. The maximum rate of PTOX was reported to be 5 e^−^ s^−1^ PSII^−1^ for PTOX2 in *C. reinhardtii* and 0.3 e^−^ s^−1^ PSII^−1^ in tomato while the maximal rate of photosynthesis is approximately 150 e^−^ s^−1^ PSII^−1^ (Nawrocki et al., 2015). However in plants exposed to stress, PTOX activity can account for 30% of the PSII activity (Stepien and Johnson, 2009). The *in vitro* enzyme activity of PTOX is high when substrate concentrations are saturating (up to 19.01 ± 1.1 μmol O_2_ mg protein^−1^ min^−1^; Yu et al., 2014). This corresponds to a turnover rate of 320 e^−^ s^−1^ PTOX^−1^ at 35°C, the optimum temperature for PTOX from rice. The discrepancy between the reported PTOX activities *in planta* and the V_*max*_ measured with the purified protein points to a mechanism that allows the regulation of PTOX activity depending on the reduction state of the electron transport chain.

Since PTOX can compete with linear and cyclic electron flow (Feilke et al., 2015) and consequently lowers NADPH, ATP production and CO_2_ fixation and potentially generates ROS, its activity must be tightly controlled. High activity is beneficial for the plant to protect the photosynthetic apparatus against photoinhibition when the electron transport chain is in a highly reduced state as it is the case under abiotic stress when the stomata are closed due to water stress or when CO_2_ fixation is limited by unfavorable temperatures. However, high PTOX activity is detrimental to high photosynthetic activity when light and CO_2_ are not limiting.

These observations have led us to postulate the following hypothesis (Figure 1) that explains the discrepancies in the literature about the safety valve function of PTOX. When stromal pH is alkaline (in high light), PTOX may become associated with the membrane giving it access to its substrate, lipophilic plastoquinol, leading to efficient oxidation of the quinol and reduction of O_2_ to H_2_O. By contrast when stroma pH becomes less alkaline (under non-saturating light conditions) PTOX may be soluble. Soluble PTOX cannot access its substrate plastoquinol that is located in the thylakoid membrane and the enzyme is effectively inactive. Activity of carotenoid biosynthesis enzymes may be regulated in a similar manner. Phytoene desaturase, which catalyzes the reaction of lipophilic phytoene to ζ-carotene, is found in the stroma both as a tetrameric membrane-bound form which has access to substrate and a soluble multi-oligomeric form in the stroma that does not (Gemmecker et al., 2015). Another example of an enzyme known to associate with the membrane in a pH-dependent manner is the violaxanthin de-epoxidase (Hager and Holocher, 1994). This enzyme associates with the thylakoid membrane when the luminal pH decreases.

**Figure 1:**
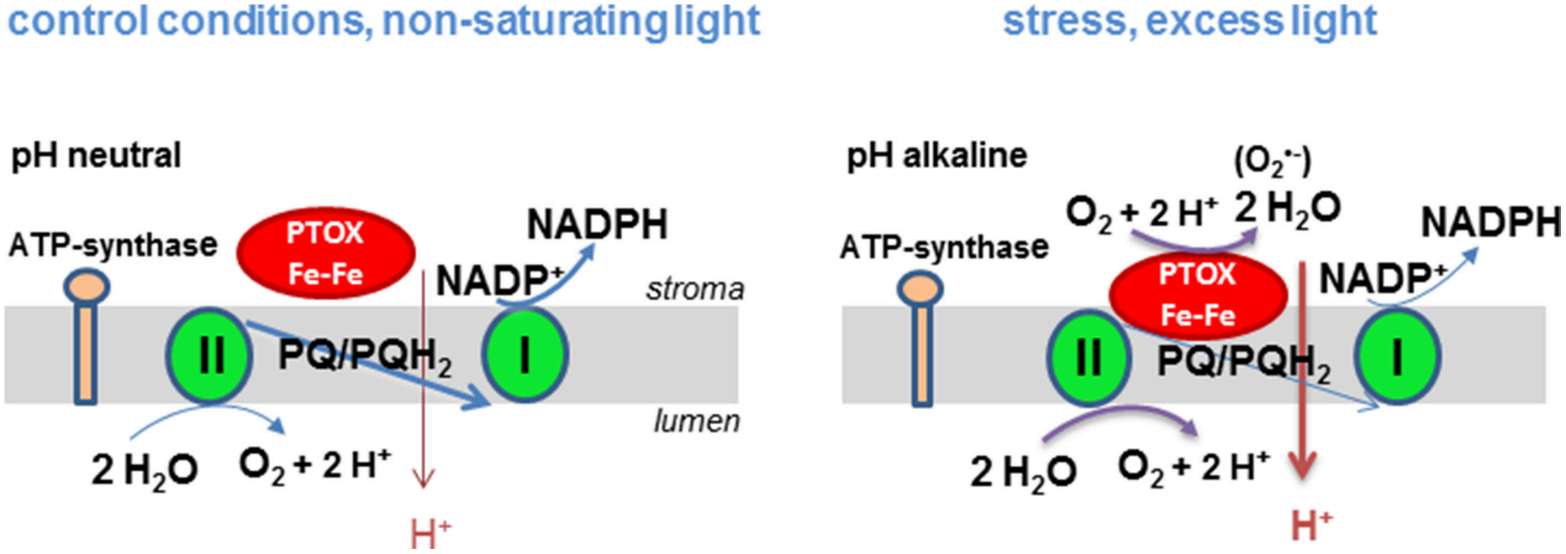
**Hypothetical model of the regulation of PTOX activity by the proton gradient in higher plants**. Under non-saturating light conditions linear electron transport between PSII and PSI takes place and a moderate proton gradient is established across the thylakoid membrane. PTOX cannot operate since it has no access to its substrate plastoquinol. At saturating light intensities linear electron transport is slowed down, the proton gradient and the plastoquinol concentration increases. The stroma gets more alkaline allowing PTOX to associate to the membrane and to catalyze the oxidation of plastoquinol. Production of ${\text{O}}_{2}^{•-}$ in a side reaction may trigger a ROS signaling pathway and thereby a stress acclimation response.

The model of pH-dependent regulation of PTOX activity by membrane association allows us to rationalize how PTOX could act as a safety valve under conditions of stress such as drought, high light and extreme temperatures when the stomata are closed and the CO_2_ assimilation rate is low and the stromal pH is alkaline. Its dissociation from the membrane at less alkaline pH would hinder its competition with the photosynthetic electron chain for its substrate plastoquinol. Chlororespiration in the dark requires membrane associated PTOX. In our model, this can only take place when a proton gradient is created in the dark by hydrolysis of ATP that is either present in the chloroplast or delivered to the chloroplast from mitochondria. Additionally, when the plastoquinone pool is highly reduced, PTOX can generate superoxide, a potential signaling mechanism that causes the expression levels of responsive genes to change allowing the plant to acclimate to changes in its environment.

## Conflict of interest statement

The authors declare that the research was conducted in the absence of any commercial or financial relationships that could be construed as a potential conflict of interest.
